# RPP30 is a novel diagnostic and prognostic biomarker for gastric cancer

**DOI:** 10.3389/fgene.2022.888051

**Published:** 2022-07-19

**Authors:** Ying Kan, Xia Lu, Lijuan Feng, Xu Yang, Huan Ma, Jianhua Gong, Jigang Yang

**Affiliations:** ^1^ Department of Nuclear Medicine, Beijing Friendship Hospital, Capital Medical University, Beijing, China; ^2^ Institute of Medicinal Biotechnology, Chinese Academy of Medical Sciences and Peking Union Medical College, Beijing, China

**Keywords:** gastric cancer, RPP30, early diagnosis, prognosis, bioinformatics analysis

## Abstract

**Objective:** This study aimed to identify the hub gene in gastric cancer (GC) tumorigenesis. A biomarker prediction model was constructed and analyzed, and protein expression in histopathological samples was verified in a validation cohort.

**Methods:** Differentially expressed genes (DEGs) were identified from GC projects in The Cancer Genome Atlas (TCGA) database. Functional enrichment analysis of DEGs was performed between the high- and low- Ribonuclease P protein subunit p30 (RPP30) expression groups. ROC analysis was performed to assess RPP30 expression to discriminate GC from normal tissues. Functional enrichment pathways and immune infiltration of DEGs were analyzed using GSEA and ssGSEA. Survival analysis and nomogram construction were performed to predict patient survival. Immunohistochemical staining of GC tissues was performed to validate RPP30 expression in GC and paracancerous samples.

**Results:** Gene expression data and clinical information of 380 cases (375 GC samples and 32 para-cancerous tissues) were collected from TCGA database. The AUC for RPP30 expression was found to be 0.785. The G alpha S signaling pathway was the most significantly enriched signaling pathway. Primary therapy outcome (*p* < 0.001, HR = 0.243, 95% CI = 0.156–0.379), age (*p* = 0.012, HR = 1.748, 95% CI = 1.133–2.698), and RPP30 expression (*p* < 0.001, HR = 2.069, 95% CI = 1.346–3.181) were identified as independent prognostic factors. As a quantitative approach, a nomogram constructed based on RPP30 expression, age, and primary therapy outcome performed well in predicting patient survival. Nineteen of the 25 tissue samples from the validation cohort showed positive RPP30 expression in GC tissues, whereas 16 cases showed negative RPP30 staining in normal tissues. The difference between the two was statistically significant.

**Conclusion:** High RPP30 expression was significantly correlated with disease progression and poor survival in GC, promoting tumorigenesis and angiogenesis *via* tRNA dysregulation. This study provides new and promising insights into the molecular pathogenesis of tRNA in GC.

## Introduction

Gastric cancer (GC) remains a major challenge in the field of oncology. It is the fifth most frequently occurring cancer and the third leading cause of cancer-related mortality (Global Burden of Disease Cancer et al., 2019). In China, epidemiological studies have shown that GC is the third most common cancer after lung cancer and liver cancer, and is characterized by high mortality and morbidity (Zhou et al., 2019). Although GC treatment protocols have improved markedly, there is still no gold standard therapy, and the 5-year overall survival (OS) rate continues to be less than 30% (Rugge et al., 2015). There are many reasons for the low survival rates, such as late-stage diagnosis, high intra-tumor heterogeneity, and chemotherapy resistance (Stahl et al., 2015). GC is characterized by high molecular and phenotypic heterogeneity. Understanding the underlying mechanism of GC carcinogenesis and progression is pivotal for the early diagnosis and improvement of survival rates.

The use of high-throughput sequencing technology has recently provided new insights into the molecular pathogenesis and prognosis prediction of GC. Hundreds of hub genes have been shown to promote tumorigenesis via different tumor-related pathways, biological processes, and molecular functions. Genetic alterations, such as aberrant DNA methylation and overexpression or downregulation of microRNAs (miRNAs), long non-coding RNAs (lncRNAs), and circulating RNAs may play a role in GC initiation and progression. However, all these plasma biomarkers are non-coding RNAs, for which improved extraction techniques, probe enrichment, and validation studies are required for clinical implementation (Smyth et al., 2020).

Ribonuclease P protein subunit p30 (RPP30), a subunit of ribonuclease P (RNase P) with a molecular weight of 30 kDa, cleaves the 5′ leader sequence from transfer RNA (tRNA) precursor molecules (Jarrous et al., 1998). RNase P, a ribonucleoprotein complex with 10 protein components and one catalytic RNA, plays important roles in genome preservation, including gene transcription, replication RNA, DNA repair, and chromatin remodeling (Lemieux et al., 2016; Jarrous, 2017; Wu et al., 2018). The catalytic RNA subunit is responsible for nuclear RNA and tRNA processing (Mondragón, 2013). RPP30 has also been demonstrated to affect the process of RNA modification in protein expression and to promote tumorigenesis in glioblastoma (Li et al., 2020a). Although RPP30 plays a pivotal role in some types of tumors, the expression of RPP30 and its biological effects in GC remain unknown.

Previous studies have confirmed that more cancer-related molecular factors may contribute to the development of GC (Sexton et al., 2020). Identification of novel biomarkers for the early diagnosis and prognosis of GC is crucial for improving treatment efficacy. In this study, we analyzed The Cancer Genome Atlas (TCGA) database to identify possible biomarkers of GC *via* bioinformatics; we further constructed a biomarker prediction model and verified the expression of proteins histopathologically in a validation cohort.

## Materials and methods

### RNA-sequencing gene expression analysis

The gene expression data (workflow type: HTSeq-FPKM and HTSeq-counts) and clinical information of 380 cases (375 GC samples and 32 para-cancerous tissues) were collected from GC projects in TCGA database (https://portal.gdc.cancer.gov/). Cases with an OS of less than 30 days (*n* = 29) were excluded. Finally, the 407 RNA-sequencing (RNA-seq) gene expression level 3 HTSeq-FPKM data of patients were transformed into transcripts per million for further analyses. Patient characteristics, including age, sex, race, TNM stage, pathologic stage, histological type, and TP53 and PIK3CA status, were recorded. Some of these data were unavailable and were treated as missing values. The study design fully satisfied the publication guidelines of TCGA.

The high and low RPP30 expression profiles (HTSeq-counts) were compared with each other within DESeq2 package (Love et al., 2014). Genes with adjusted *p-*value < 0.05 and |log2 (fold-change)| > 1.5 were considered as differentially expressed.

### Immunohistochemical assay of the validation cohort of GC patients

Twenty-five GC patients (12 men, 13 women; mean age, 61.8 years) who underwent total or subtotal gastrectomy between September 2018 and December 2019 were recruited at the Beijing Friendship Hospital of Capital Medical University. The ethic of validation cohort was approved by Research Ethics Committee of Beijing Friendship Hospital (NO. 2018-92-045-01). All subjects provided written informed consent in accordance with the Declaration of Helsinki.

Both GC and paracancerous tissues were prepared using formalin-fixed and paraffin-embedded sections. Sodium citrate buffer (pH 6.0) and 3% hydrogen peroxide were used for antigen retrieval and endogenous peroxidase clearance, respectively. The sections were then blocked with 5% bovine serum albumin and incubated overnight (16–18 h) with primary antibody (Anti-RPP30, Solarbio Life Science, Beijing, China). The reaction products were stained with 3,3-diaminobenzidine and counterstained with hematoxylin. Tissue slides were scanned using a Pannoramic MIDI automated slide scanner (3D Histech, Munich, Germany). Immunohistochemical analysis of RPP30 expression was performed by examining three to five random fields under ×400 magnification in the selected area of each slide. The expression levels of RPP30 protein were classified as positive (RPP30 staining positive cells >10%) or negative (RPP30 staining positive cells <10%). Two investigators who were unaware of the final diagnosis calculated the number of cells.

### Gene set enrichment analysis

GSEA was performed using the ClusterProfiler package (Yu et al., 2012) to elucidate the difference between the high and low RPP30 expression groups (RPP30^high^ and RPP30^low^, respectively). The RPP30 expression level was regarded as a phenotype, and all gene set permutations were performed 1,000 times for each analysis. Any function or pathway with adjusted *p*-value < 0.05, FDR q-value < 0.25, and absolute value of the normalized enrichment score (NES) > 1 were statistically significant.

### Correlation between RPP30 expression and immune infiltration

Single-sample GSEA (ssGSEA) from the GSVA package (Barbie et al., 2009) was applied to quantify the relative tumor infiltration levels of 24 immune cells by obtaining the expression levels of genes in published gene lists (Bindea et al., 2013).

The correlation between RPP30 expression and immune cell infiltration was analyzed using Spearman’s correlation, and the infiltration of immune cells between the RPP30^high^ and RPP30^low^ groups was analyzed using the Wilcoxon rank-sum test.

### Prognostic model generation and prediction

Survival analysis between the RPP30^high^ and RPP30^low^ groups was performed in GC patients. The primary endpoint was OS, and the secondary endpoint was progression-free survival (PFS). The follow-up duration was estimated using the Kaplan-Meier method, and differences between survival curves were examined using the log-rank test. Univariate Cox proportional hazards regression was used to estimate the hazard ratio (HR) for OS and PFS. The significant variables, as determined via univariate analysis, were included in the multivariate analysis. Multivariate Cox regression analysis was used to evaluate the optimal model.

A nomogram was constructed to predict the individualized survival probability of GC patients. The risk score was calculated as the sum of scores for each parameter. A calibration plot was used to evaluate the prediction accuracy of the nomogram based on the prognostic model. All statistical tests were two-tailed, and *p*-values < 0.05 were considered statistically significant.

### Statistical analysis

All statistical analyses were performed using R software (http://www.r-project.org/, version 3.6.2). The cut-off value of RPP30 expression was determined by the median value and used for grouping into the RPP30^high^ and RPP30^low^ groups. The Wilcoxon rank-sum test was used to analyze the expression of RPP30 in normal and tumor samples. Kruskal–Wallis and Spearman’s correlation tests were used to evaluate the relationship between RPP30 expression and GC clinicopathological features.

## Results

### Demographic characteristics and RPP30 expression

The clinical characteristics of GC patients in TCGA database, including sex, age, race, TNM stage, histological type and grade, clinical presentation, and PIK3CA status, were obtained (Supplementary Table S1). A total of 134 female and 241 male patients were included in the study.

Considering the TNM pathologic stage, 53 cases (14.1%) were stage I, 111 (29.6%) were stage II, 150 (40.0%) were stage III, and 38 (10.1%) were stage IV. Regarding histological grade, 10 cases (2.6%) were well differentiated (G1 group), 137 cases (36.5%) were moderately differentiated (G2 group), and 219 cases (58.4%) were poorly differentiated (G3 group). According to PIK3CA status, 59 cases (15.7%) were mutants, and 313 cases (83.4%) were wild-type.

### Potential role of RPP30 in regulating the progression of GC

**FIGURE 1:**
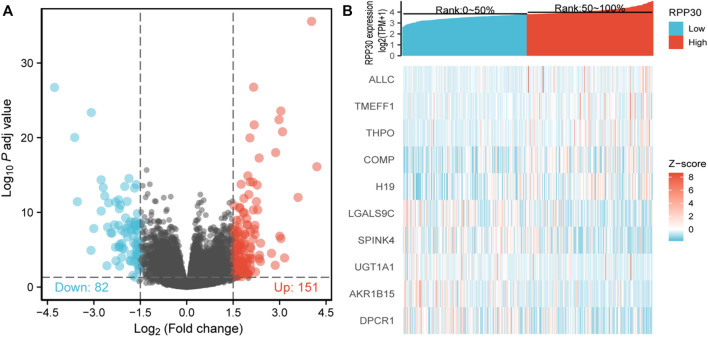
Identification of DEGs between RPP30^high^ and RPP30^low^ groups. **(A)** Volcano plot of DEG profiles between RPP30^high^ and RPP30^low^ groups. A total of 233 DEGs were obtained, of which 151 were upregulated and 82 were downregulated. **(B)** Heatmap of GO analysis showing the co-expression of differential gene profiles in TCGA between RPP30^high^ and RPP30^low^ groups. Red indicates upregulated genes; blue indicates downregulated genes; each row indicates each gene expression in different samples, whereas each column indicates the expression of all genes in each sample.

To elucidate whether RPP30 plays a role in prompting GC, TCGA RNA-seq data analysis was performed to compare DEGs between the RPP30^high^ and RPP30^low^ groups. A total of 151 upregulated and 82 downregulated genes were identified in the RPP30^high^ group (using the RPP30^low^ group as reference). DEG expression is shown as a volcano map and heat map in Figure 1.

**FIGURE 2:**
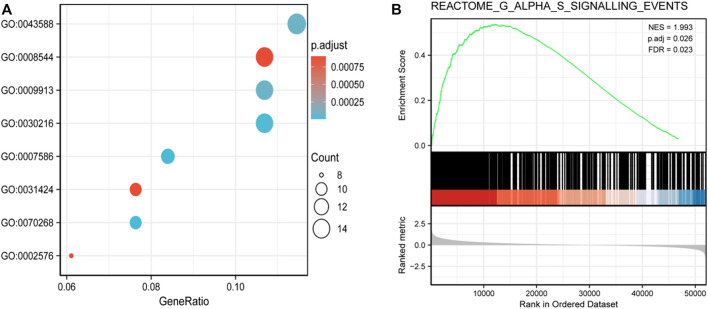
Functional enrichment analysis of DEGs between RPP30^high^ and RPP30^low^ groups of GC in TCGA. **(A)** Enriched GO terms in the biological process category. The *x*-axis represents the proportion of DEGs, and the *y*-axis represents different categories. In this category, skin development (GO: 0043588), epidermis development (GO: 0008544), epidermal cell differentiation (GO:0009913), and keratinocyte differentiation (GO: 0030216) were primarily enriched. **(B)** GSEA results showed that the G alpha S signaling pathway was the most enriched. (NES, normalized enriched score; *P*. adj, adjusted *p*-value; FDR, false discovery rate; gene sets with *P*. adj <0.05, FDR q-value < 0.25, and |NES| > 1 are considered as significant).

Gene Ontology (GO) and Kyoto Encyclopedia of Genes and Genomes functional enrichment analyses were performed to further understand the functional implications of RPP30 in GC. Biological processes were primarily enriched in skin development (GO: 0043588), epidermal development (GO: 0008544), epidermal cell differentiation (GO:0009913), and keratinocyte differentiation (GO: 0030216). The GSEA results showed that several pathways and biological processes were differentially enriched in RPP30 in relation to GC, including the G alpha S signaling pathway, neuronal system, and olfactory transduction. Based on the NES score, the most significantly enriched signaling pathway was the G alpha S signaling pathway. The results suggested a link between the aberrant *RPP30* gene and immune response (Figure 2).

### Correlation between RPP30 expression and immune infiltration

**FIGURE 3:**
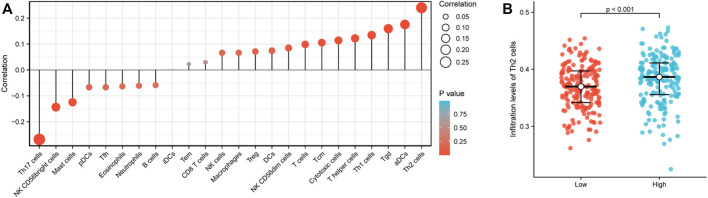
RPP30 expression level was associated with immune infiltration in the GC microenvironment. **(A)** Correlation between the marker gene of 24 immune cells and RPP30 expression level is shown in the lollipop chart. The size of the dots shows the absolute value of Spearman’s correlation coefficient (r). Larger dots indicate higher correlation coefficients. RPP30 expression was positively correlated with the abundances of immunocytes (Th2 cells, activated dendritic cells, Th1 cells, and helper T cells) and negatively correlated with the presence of Th17 cells. **(B)** Difference between RPP30^high^ and RPP30^low^ groups in terms of Th2 cell infiltration.

The correlation between the expression level of RPP30 and immune cell infiltration is depicted as a lollipop chart (Figure 3A). RPP30 expression was positively correlated with the abundance of immunocytes (Th2 cells, activated dendritic cells, Th1 cells, and helper T cells) and negatively correlated with that of Th17 cells. In comparison to the low expression of RPP30, the high expression of RPP30 was associated with increased Th2 cell infiltration in the tumor microenvironment (*p* < 0.001; Figure 3B).

### Association with RPP30 expression and clinicopathological variables

**FIGURE 4:**
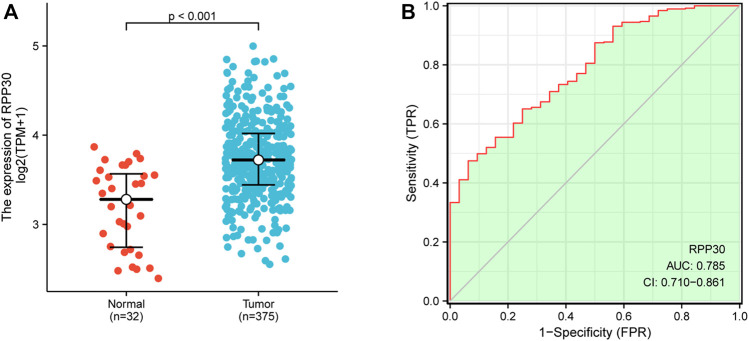
RPP30 expression between normal and GC tumor samples in TCGA database. **(A)** RPP30 was significantly upregulated in GC (*p* < 0.001). **(B)** ROC analysis of RPP30 expression showed high diagnostic efficiency in discriminating between tumor and normal tissues. The AUC of RPP30 in the diagnosis of GC was 0.785.

**FIGURE 5:**
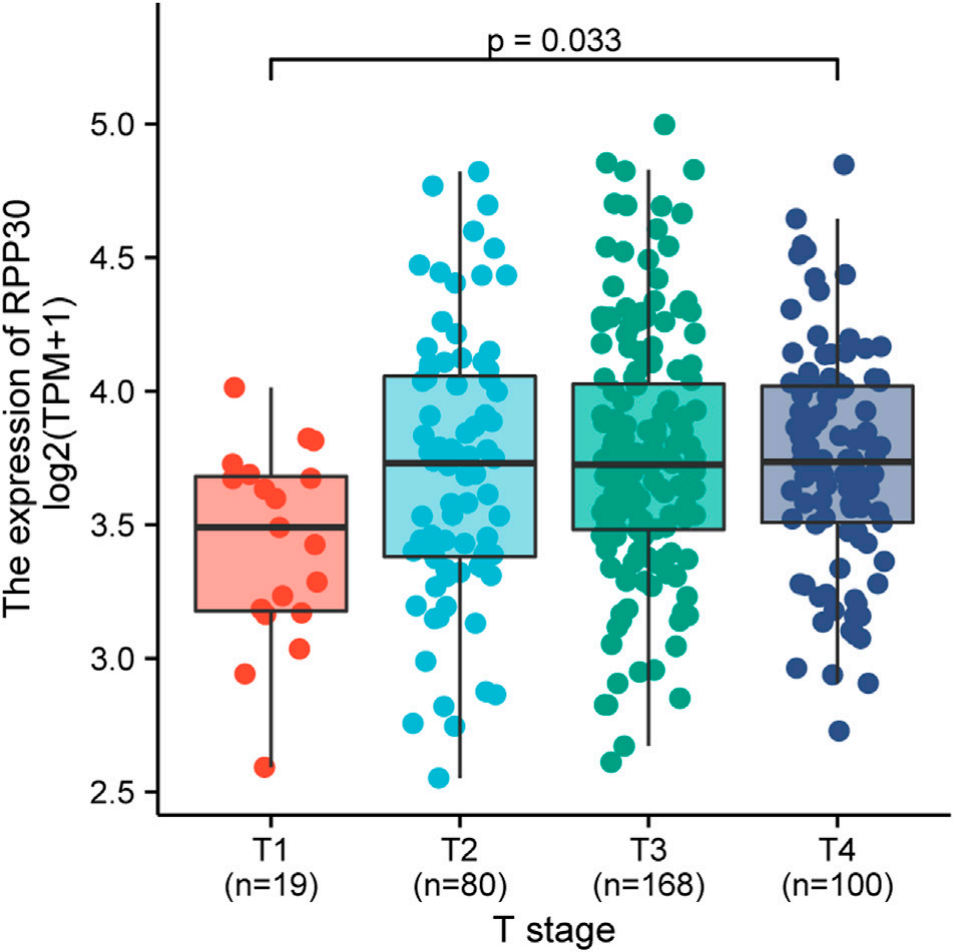
Relationship between RPP30 expression and T-stage of TCGA in GC. The results showed that RPP30 expression was significantly correlated with T-stage (*p* = 0.033). Bonferroni correction between each group also showed significance.

There was a significant statistical difference in RPP30 expression between tumor and paracancerous normal tissues (*p* < 0.001), and a higher expression of RPP30 was observed in tumor tissues (Figure 4A). Furthermore, we performed ROC analysis to calculate the diagnostic performance of RPP30 expression. The ROC curve showed that the AUC of RPP30 expression for distinguishing tumors from normal tissues was 0.785, indicating a high diagnostic accuracy for GC (Figure 4B). RPP30 expression was significantly correlated with the T stage of GC (Figure 5). These results provided a theoretical basis for the use of RPP30 as a biomarker in the early diagnosis of GC.

A total of 375 samples with RPP30 expression data were analyzed from TCGA database. Logistic regression analysis revealed that the expression of RPP30 was associated with poor clinicopathological characteristics (Supplementary Table S2), including histological grade (*p* = 0.018, OR = 1.66, 95% CI = 1.09–2.54) and PIK3CA status (*p* = 0.031, OR = 1.87, 95% CI = 1.07–3.37).

### Survival analysis

Kaplan-Meier survival analysis was performed to evaluate the association between RPP30 expression and GC prognosis. High RPP30 expression was more strongly associated with a worse prognosis than low RPP30 expression. Univariate Cox regression analysis revealed that high RPP30 expression was correlated with shorter OS (HR = 1.53, 95% CI = 1.10–2.14, *p* = 0.012). Univariate analysis also revealed that T stage (*p* = 0.011), N stage (*p* = 0.002), M stage (*p* = 0.004), pathological stage (*p* < 0.001), primary therapy outcome (*p* < 0.001), residual tumor (*p* < 0.001), age (*p* = 0.005), and RPP30 expression (*p* = 0.012) were significantly correlated with OS. These parameters were then included in the multivariate Cox regression model, which revealed that the primary therapy outcome (*p* < 0.001, HR = 0.243, 95% CI = 0.156–0.379), age (*p* = 0.012, HR = 1.748, 95% CI = 1.133–2.698), and RPP30 expression (*p* < 0.001, HR = 2.069, 95% CI = 1.346–3.181) could serve as independent prognostic factors for GC (Supplementary Table S3).

### Construction and validation of the prognostic model based on RPP30 expression and clinicopathological factors

**FIGURE 6:**
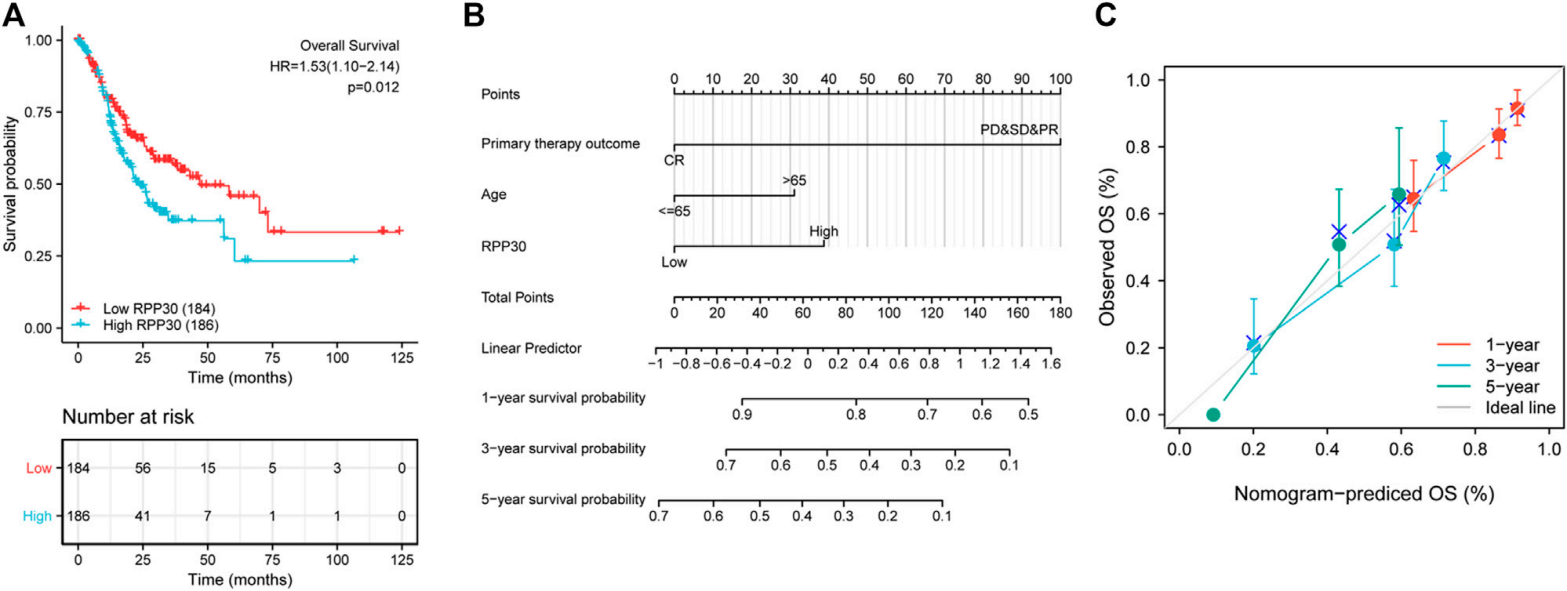
Validation of RPP30-based nomogram for GC patients. **(A)** Effect of RPP30 expression on the OS of GC patients in TCGA cohort. The results showed that higher RPP30 expression was associated with poor OS (HR = 1.53 [1.10–2.14], *p* = 0.012). **(B)** Nomogram for the prediction of 1-, 3-, and 5-years OS of GC patients. The C-index was 0.704 (95% CI = 0.680–0.728). **(C)** The calibration plot of the nomogram indicated a good agreement between the prediction and the ideal line.

To establish a quantitative approach to GC prognosis, we constructed a prognostic nomogram model based on the results of multivariate Cox regression analysis involving RPP30 and independent clinicopathological risk factors. The concordance index (C-index) for the nomogram was 0.704 (95% CI = 0.680–0.728). The total score was the sum of the scores for each variable. The probability of GC patient survival at 1, 3, and 5 years was determined by drawing a vertical line along the total points (Figure 6). The calibration plot demonstrated that the bias-corrected line was close to the ideal line, which indicated good agreement between the observation and prediction. These findings suggested that the nomogram is a better model for predicting OS in GC patients than individual prognostic factors.

### RPP30 expression affects the prognosis of GC at different clinicopathological statuses

**FIGURE 7:**
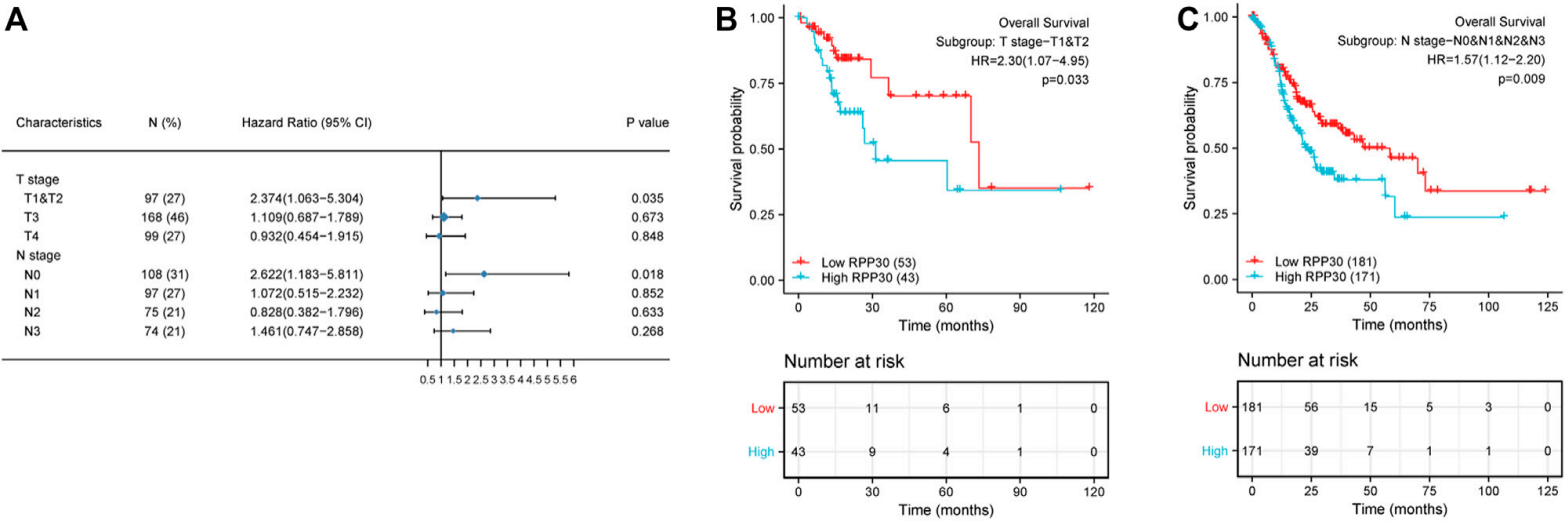
Subgroup survival analysis of clinical characteristics of GC. **(A)** High RPP30 expression was correlated with worse OS in the T1 and T2 stages of GC, but not in the T3 and T4 stages. In the N stages, high RPP30 expression was associated with worse OS in the N0, but not in the N1 - N3 stages of GC. **(B)** The K-M plot of OS showed that high RPP30 expression had higher HR value (HR = 2.3, *p* = 0.033) in subgroups of T1 and T2 stages. **(C)** The hazard ratio in the high-RPP30 expression group was 0.57 times higher than that in the low expression group (*p* = 0.009).

To further understand the mechanism of RPP30 expression, we investigated the relationship between RPP30 expression and clinicopathological status. Univariate Cox analysis revealed that RPP30 expression was associated with poor OS in the T and N stages, especially in the T1, T2, and N0 stages. This indicated that in the T1, T2, and N0 stages, high RPP30 expression was associated with worse OS (Figure 7). These results suggested that RPP30 expression levels affect the prognosis of GC with different clinicopathological statuses, and that the early detection of RPP30 expression is pivotal for the prognosis of GC patients.

### Validation of RPP30 expression in GC tissues

**FIGURE 8:**
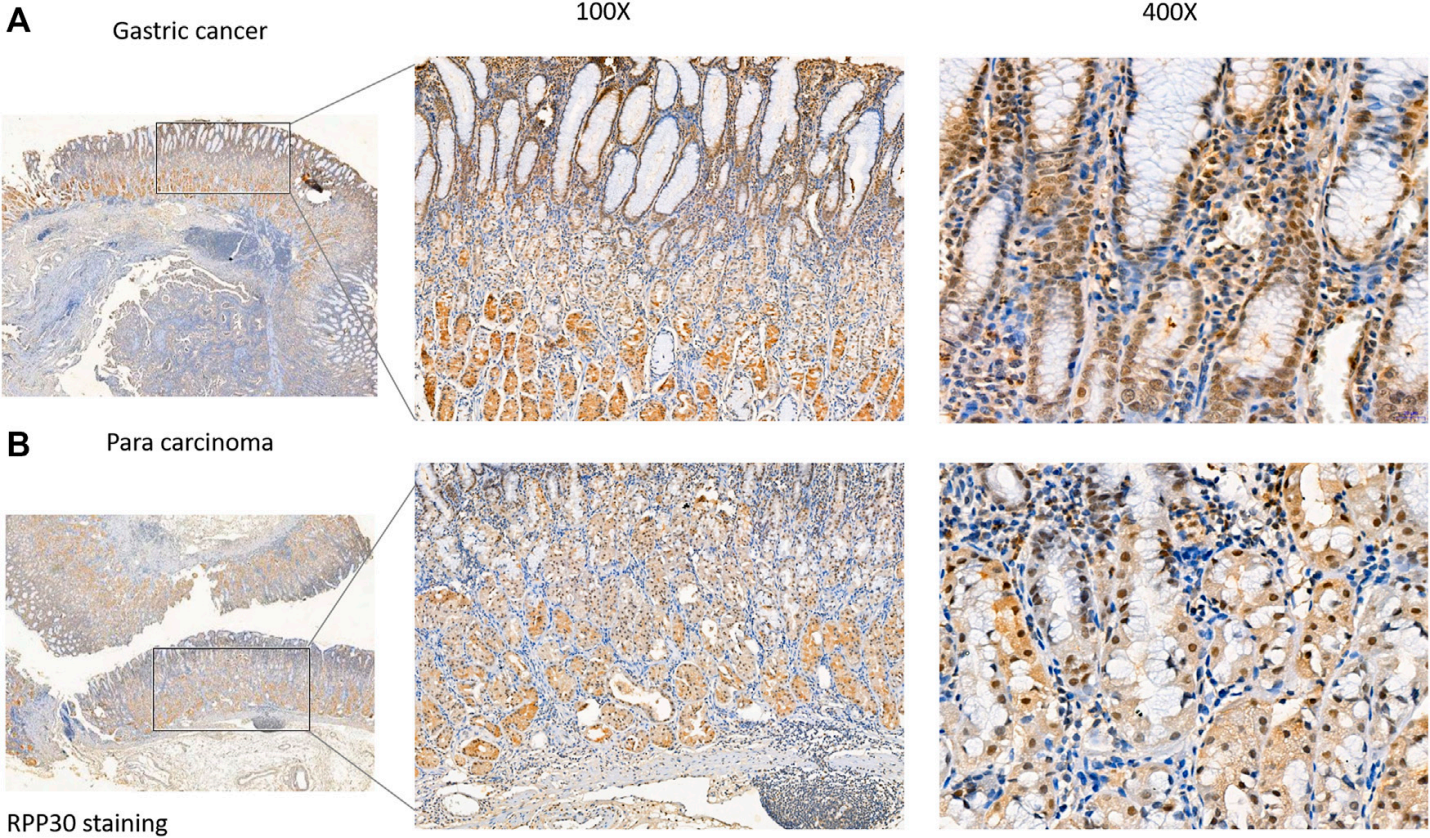
RPP30 protein expression in GC and para-cancerous tissues assessed via immunohistochemical staining. **(A)** RPP30 expression was found in GC specimens, especially in the nucleus of the glandular epithelium. **(B)** RPP30 protein expression was negative in para-cancerous tissues.

RPP30 expression was investigated using immunohistochemistry in a validation cohort of 25 GC tissue samples. Of the 25 specimens, 19 showed positive RPP30 expression in GC tissue and 16 showed negative RPP30 expression in normal tissues (Supplementary Table S4). The difference between the two was significant. The RPP30 protein was primarily distributed in the nucleus and cytoplasm (Figure 8). The expression of RPP30 protein was significantly higher in GC tissues than in para-cancerous tissues.

## Discussion

GC is the fifth most common malignancy worldwide. It has become the third most common cancer-related cause of death because it is usually diagnosed when the cancer reaches an advanced stage. Therefore, it is essential to explore the molecular mechanisms of GC for its early diagnosis and prognosis. Various GC-related miRNAs, lncRNAs, cytokines, and proteins are disordered and associated with the early diagnosis or prognosis of GC (Wu et al., 2019). However, the sensitivity of these biomarkers for discriminating between early-stage GC and healthy subjects is low (Necula et al., 2019). The expression and function of RPP30 in gliomas has been previously reported (Li et al., 2020a). RPP30-related proteins were primarily enriched in the cancer-related pathways. To our knowledge, the expression of RPP30 and its potential prognostic impact on GC remain unexplored. In this study, bioinformatics analysis of high-throughput RNA-seq data from TCGA demonstrated that high RPP30 expression in GC was associated with advanced clinical pathological characteristics, survival time, and poor prognosis.

RPP30, a subunit of RNase P, a ribozyme involved in pre-tRNA processing, forms mature tRNAs that bind to amino acids and further regulate protein expression. Our results showed that RPP30 was highly expressed in GC tissues compared to normal tissues and showed high diagnostic accuracy for GC. RPP30 was primarily enriched in epidermal development, cell differentiation, and keratinocyte differentiation, and was differentially enriched in the RPP30-high expression phenotype. Previous studies have shown that the differentiation of keratinocytes plays an important role in the differentiation of normal gastric epithelial cells and affects the function of parietal cells (Matsunobu et al., 2006). RPP30 was significantly enriched in the G alpha S signaling pathway and increased cAMP levels (Loffler et al., 2008; Wehbe et al., 2020), and it was related to the histopathology of GC. Our immunohistochemistry results showed that RPP30 expression was significantly higher in GC tissues than in paracancerous tissues and correlated with clinicopathological features. These results provide a theoretical basis for the early diagnosis of GC.

RPP30 affects tRNA processing, transcription replication, DNA repair, and replication fork stalling (Molla-Herman et al., 2015; Wu et al., 2018); regulates protein expression; enriches cancer-related pathways, leading to tumorigenesis; and eventually promotes the proliferation, metastasis, and invasiveness of cancer cells (Huang et al., 2018). Multiple large-scale genomic studies have shown that altering the co-transcriptional and post-transcriptional regulation of gene expression during RNA processing, including the splicing, transport, editing, and decay of messenger RNA, can initiate tumorigenesis and cancer maintenance (Obeng et al., 2019). Dysregulation of tRNA in tumor pathogenesis has been confirmed in breast cancer, lung cancer, and melanoma; however, relevant studies on GC are still lacking (Huang et al., 2018). In addition, high RPP30 expression was shown to be correlated with the poor prognosis of GC at the T1 to T2 and N0 stages, with the highest HR for poor OS when RPP30 was highly expressed in GC tissues. These findings strongly suggest that RPP30 expression is a powerful indicator of GC prognosis in these subsets. For more accurate prognosis prediction, nomograms have been developed that show better performance than conventional staging systems (Li et al., 2020b; Wang et al., 2020). Our nomogram included three parameters available from clinical records and tissue specimens. As previously reported, age is an independent prognostic factor, and older age is associated with poorer prognosis (Schlesinger-Raab et al., 2016). In the revised RECIST guidelines, response to chemotherapy has been shown to be associated with OS (Eisenhauer et al., 2009). Primary therapy outcome with complete response was associated with a better prognosis than partial response. These results are consistent with our findings. Highly fitted calibration plots demonstrated that the nomogram performed well in predicting the 3- or 5-years survival of GC patients.

Although the present study improved our understanding of the relationship between RPP30 expression and GC, some limitations remain. First, due to the limitations of the study design, we will further examine the additional RPP30-relevant signaling pathways and investigate the mechanism of RPP30 in GC tumorigenesis by experimental studies. Second, although public databases are multicenter, retrospective studies have limitations. In the future, prospective studies should be performed to avoid selection bias.

Our study demonstrated that high RPP30 expression was significantly correlated with tumor progression and poor survival in GC, which might promote tumorigenesis and angiogenesis via tRNA dysregulation. This study provides new insights into the molecular pathogenesis of tRNA in GC.

## Data Availability

The datasets presented in this study can be found in online repositories. The names of the repository/repositories and accession number(s) can be found in the article/Supplementary Material.
