# Supplementary material for: RPP30 is a novel diagnostic and prognostic biomarker for gastric cancer
Source: Front Genet. 2022 Jul 19;13:888051. doi: 10.3389/fgene.2022.888051 (PMC9343801; doi:10.3389/fgene.2022.888051)
Supplement: Supplementary file 1 [file Table1.DOCX]

Table 1. Demographic characteristics of gastric cancer based on TCGA database according to RPP30 expression.

| **Based**  **characteristics** | **level** | **Low expression of RPP30(N=188)** | **High expression of RPP30 (N=187)** | ***p*-value** | **test** |
| --- | --- | --- | --- | --- | --- |
| Gender (n, %) | Female | 65(34.6%) | 69(36.9%) | 0.718 |  |
|  | Male | 123(65.4%) | 118(63.1%) |  |  |
| Age (n, %) | <=65 | 74(40.0%) | 90(48.4%) | 0.128 |  |
|  | >65 | 111(60.0%) | 96(51.6%) |  |  |
| Age (median [IQR]) |  | 68.00[60.00,74.00] | 66.00[57.00,72.75] | 0.062 | Nonnorm |
| Race (n, %) | Asian | 37(23.0%) | 37(22.8%) | 0.326 | exact |
|  | Black or African American | 8(5.0%) | 3(1.9%) |  |  |
|  | White | 116(72.0%) | 122(75.3%) |  |  |
| T stage (n, %) | T1 | 15(8.1%) | 4(2.2%) | 0.081 | exact |
|  | T2 | 40(21.5%) | 40(22.1%) |  |  |
|  | T3 | 84(45.2%) | 84(46.4%) |  |  |
|  | T4 | 47(25.3%) | 53(29.3%) |  |  |
| N stage (n, %) | N0 | 59(31.9%) | 52(30.2%) | 0.930 |  |
|  | N1 | 50(27.0%) | 47(27.3%) |  |  |
|  | N2 | 40(21.6%) | 35(20.3%) |  |  |
|  | N3 | 36(19.5%) | 38(22.1%) |  |  |
| M stage, (n, %) | M0 | 165(92.7%) | 165(93.2%) | 1.000 |  |
|  | M1 | 13(7.3%) | 12(6.8%) |  |  |
| Pathologic stage (n,%) | Stage I | 32(17.7%) | 21(12.3%) | 0.550 |  |
|  | Stage II | 54(29.8%) | 57(33.3%) |  |  |
|  | Stage III | 76(42.0%) | 74(43.3%) |  |  |
|  | Stage IV | 19(10.5%) | 19(11.1%) |  |  |
| Anatomic neoplasm subdivision (n, %) | Antrum/Distal | 69(37.3%) | 69(39.2%) | 0.020 |  |
|  | Cardia/Proximal | 24(13.0%) | 24(13.6%) |  |  |
|  | Fundus/Body | 59(31.9%) | 71(40.3%) |  |  |
|  | Gastroesophageal Junction | 31(16.8%) | 10(5.7%) |  |  |
|  | Other | 2(1.1%) | 2(1.1%) |  |  |
| Histological type (n, %) | Diffuse Type | 32(17.0%) | 31(16.7%) | 0.390 | exact |
|  | Mucinous Type | 14(7.4%) | 5(2.7%) |  |  |
|  | Not Otherwise Specified | 98(52.1%) | 109(58.6%) |  |  |
|  | Papillary Type | 3(1.6%) | 2(1.1%) |  |  |
|  | Signet Ring Type | 6(3.2%) | 5(2.7%) |  |  |
|  | Tubular Type | 35(18.6%) | 34(18.3%) |  |  |
| Residual tumor (n, %) | R0 | 157(90.2%) | 141(91.0%) | 0.834 |  |
|  | R1 | 9(5.2%) | 6(3.9%) |  |  |
|  | R2 | 8(4.6%) | 8(5.2%) |  |  |
| Histologic grade (n, %) | G1 | 7(3.8%) | 3(1.6%) | 0.046 | exact |
|  | G2 | 78(42.4%) | 59(32.4%) |  |  |
|  | G3 | 99(53.8%) | 120(65.9%) |  |  |
| Primary therapy outcome (n, %) | CR | 118(72.0%) | 113(73.9%) | 0.976 | exact |
|  | PD | 35(21.3%) | 30(19.6%) |  |  |
|  | PR | 2(1.2%) | 2(1.3%) |  |  |
|  | SD | 9(5.5%) | 8(5.2%) |  |  |
| Reflux history (n, %) | No | 97(80.2%) | 78(83.9%) | 0.605 |  |
|  | Yes | 24(19.8%) | 15(16.1%) |  |  |
| Antireflux treatment (n, %) | No | 69(73.4%) | 73(85.9%) | 0.061 |  |
|  | Yes | 25(26.6%) | 12(14.1%) |  |  |
| Barretts esophagus (n, %) | No | 115(92.7%) | 78(92.9%) | 1.000 |  |
|  | Yes | 9(7.3%) | 6(7.1%) |  |  |
| TP53 status (n, %) | Mut | 84(44.9%) | 88(47.6%) | 0.683 |  |
|  | WT | 103(55.1%) | 97(52.4%) |  |  |
| PIK3CA status (n, %) | Mut | 22(11.8%) | 37(20.0%) | 0.042 |  |
|  | WT | 165(88.2%) | 148(80.0%) |  |  |

**Abbreviation:** Nonnorm: Non-normal distribution and used Wilcoxon rank sum test.

exact: Fisher’s exact test
