# Supplementary material for: RPP30 is a novel diagnostic and prognostic biomarker for gastric cancer
Source: Front Genet. 2022 Jul 19;13:888051. doi: 10.3389/fgene.2022.888051 (PMC9343801; doi:10.3389/fgene.2022.888051)
Supplement: Supplementary file 2 [file Table2.DOCX]

Table2. RPP30 expression associated with clinical-pathological feature

| **Characteristics** | **Number** | **Odds Ratio (OR)** | ***p-* value** |
| --- | --- | --- | --- |
| T stage (T3&T4 vs. T1&T2) | 367 | 1.31(0.82-2.08) | 0.257 |
| N stage (N1&N2&N3 vs. N0) | 357 | 1.08(0.69-1.70) | 0.735 |
| M stage (M1 vs. M0) | 355 | 0.92(0.40-2.09) | 0.847 |
| Pathologic stage (Stage III&Stage IV vs. Stage I&Stage II) | 352 | 1.08(0.71-1.64) | 0.721 |
| Histological type (Diffuse Type vs. Tubular Type) | 132 | 1.00(0.50-1.98) | 0.994 |
| Histologic grade (G3 vs. G1&G2) | 366 | 1.66(1.09-2.54) | 0.018 |
| Primary therapy outcome (CR vs. PD&SD&PR) | 317 | 1.10(0.67-1.81) | 0.703 |
| Residual tumor (R1&R2 vs. R0) | 329 | 0.92(0.43-1.93) | 0.819 |
| TP53 status (Mut vs. WT) | 372 | 1.11(0.74-1.67) | 0.609 |
| PIK3CA status (Mut vs. WT) | 372 | 1.87(1.07-3.37) | 0.031 |
