# Supplementary material for: RPP30 is a novel diagnostic and prognostic biomarker for gastric cancer
Source: Front Genet. 2022 Jul 19;13:888051. doi: 10.3389/fgene.2022.888051 (PMC9343801; doi:10.3389/fgene.2022.888051)
Supplement: Supplementary file 3 [file Table3.DOCX]

Table 3. Association with overall survival and clinical pathological characteristics in TCGA database using Cox regression.

| **Characteristics** | **Total(N)** | **Univariate analysis** | | **Multivariate analysis** | |
| --- | --- | --- | --- | --- | --- |
|  |  | **HR (95% CI)** | **P value** | **HR (95% CI)** | **P value** |
| T stage (T3&T4 vs. T1&T2) | 362 | 1.719(1.131-2.612) | 0.011 | 1.172(0.624-2.202) | 0.621 |
| N stage (N1&N2&N3 vs. N0) | 352 | 1.925(1.264-2.931) | 0.002 | 1.511(0.705-3.240) | 0.289 |
| M stage (M1 vs. M0) | 352 | 2.254(1.295-3.924) | 0.004 | 0.823(0.323-2.097) | 0.683 |
| Pathologic stage (Stage III&Stage IV vs. Stage I&Stage II) | 347 | 1.947(1.358-2.793) | <0.001 | 1.290(0.671-2.480) | 0.446 |
| Histologic grade (G3 vs. G1&G2) | 361 | 1.353(0.957-1.914) | 0.087 | 1.495(0.953-2.346) | 0.080 |
| Histological type (Diffuse Type vs. Tubular Type) | 132 | 1.077(0.620-1.872) | 0.793 |  |  |
| Primary therapy outcome (CR vs. PD&SD&PR) | 313 | 0.237(0.163-0.344) | <0.001 | 0.243(0.156-0.379) | <0.001 |
| Residual tumor (R1&R2 vs. R0) | 325 | 3.445(2.160-5.494) | <0.001 | 1.744(0.898-3.387) | 0.100 |
| Age (>65 vs. <=65) | 367 | 1.620(1.154-2.276) | 0.005 | 1.748(1.133-2.698) | 0.012 |
| Race (Asian&Black or African American vs. White) | 320 | 0.801(0.515-1.247) | 0.326 |  |  |
| Gender (Male vs. Female) | 370 | 1.267(0.891-1.804) | 0.188 |  |  |
| Anatomic neoplasm subdivision (Fundus/Body vs. Antrum/Distal) | 267 | 0.965(0.651-1.430) | 0.858 |  |  |
| Reflux history (Yes vs. No) | 213 | 0.582(0.291-1.162) | 0.125 |  |  |
| Antireflux treatment (Yes vs. No) | 179 | 0.756(0.422-1.353) | 0.346 |  |  |
| Barretts esophagus (Yes vs. No) | 207 | 0.892(0.326-2.441) | 0.824 |  |  |
| TP53 status (Mut vs. WT) | 367 | 0.865(0.621-1.205) | 0.392 |  |  |
| PIK3CA status (Mut vs. WT) | 367 | 0.623(0.370-1.048) | 0.075 | 0.553(0.304-1.006) | 0.052 |
| RPP30 (High vs. Low) | 370 | 1.533(1.100-2.136) | 0.012 | 2.069(1.346-3.181) | <0.001 |
