# Supplementary material for: RPP30 is a novel diagnostic and prognostic biomarker for gastric cancer
Source: Front Genet. 2022 Jul 19;13:888051. doi: 10.3389/fgene.2022.888051 (PMC9343801; doi:10.3389/fgene.2022.888051)
Supplement: Supplementary file 4 [file Table4.DOCX]

Table 4. Baseline characteristics of gastric cancer in validation cohort

| **Characteristics** | | **Gastric cancer (N=25)** |
| --- | --- | --- |
| Gender (n, %) | Female | 6(24.0%) |
|  | Male | 19(79.1%) |
| Age (n, %) | <=65 | 4(16.0%) |
|  | >65 | 21(84.0%) |
| Age (median [IQR]) |  | 67[51,73] |
| T stage (n, %) | T1 | 4(16.0%) |
|  | T2 | 5(20.0%) |
|  | T3 | 9(36.0%) |
|  | T4 | 7(28.0%) |
| N stage (n, %) | N0 | 3(12.0%) |
|  | N1 | 6(24.0%) |
|  | N2 | 10(40.0%) |
|  | N3 | 6(24.0%) |
| M stage, (n, %) | M0 | 21(84.0%) |
|  | M1 | 4(16.0%) |
| Pathologic stage (n,%) | Stage I | 4(16.0%) |
|  | Stage II | 9(36.0%) |
|  | Stage III | 8(32.0%) |
|  | Stage IV | 4(16.0%) |
| Anatomic neoplasm subdivision (n, %) | Antrum/Distal | 8(32.0%) |
|  | Cardia/Proximal | 5(20.0%) |
|  | Fundus/Body | 9(36.0%) |
|  | Gastroesophageal Junction | 3(12.0%) |
| Histological type (n, %) | Diffuse Type | 1(4.0%) |
|  | Mucinous Type | 19(76.0%) |
|  | Papillary Type | 1(4.0%) |
|  | Signet Ring Type | 4(16.0%) |
| Histologic grade (n, %) | G1 | 1(4.0%) |
|  | G2 | 10 (40%) |
|  | G3 | 14 (56.0%) |
| TP53 status (n, %) | Mut | 5(20.0%) |
|  | WT | 20(80.0%) |
| PIK3CA status (n, %) | Mut | 3(12.0%) |
|  | WT | 22(88.0%) |
| RPP30 expression | Positive | 19(76.0%) |
|  | Negative | 6(24.0%) |
